# Risk Factors For Recurrent Stroke After Coronary Artery Bypass Grafting

**DOI:** 10.1186/1749-8090-6-157

**Published:** 2011-11-23

**Authors:** Li Cao, Qin Li, Qi Bi, Qin-Jun Yu

**Affiliations:** 1 Department of Neurology, Beijing An Zhen Hospital of the Capital Medical University, Beijing, China; 2 Department of Cardiac Surgery, Beijing An Zhen Hospital of the Capital Medical University, Beijing, China; 3 Department of Anesthesiology, Cardiovascular Institute and Fuwai Hospital, Chinese Academic Medical Science and Peking Union Medical College, Beijing, China

**Keywords:** stroke, coronary artery bypass grafting, risk factors

## Abstract

**Objectives:**

Preventing stroke after coronary artery bypass grafting (CABG) remains a therapeutic goal, due in part to the lack of identifiable risk factors. The aim of this study, accordingly, was to identify risk factors in CABG patients with a previous history of stroke.

**Methods:**

Patients with a history of stroke who underwent CABG at Beijing An Zhen hospital from January 2007 to July 2010 were selected (n = 430), and divided into two groups according to the occurrence of postoperative stroke. Pre-operative and post-operative data were retrospectively collected and analyzed by univariate and multivariate logistic regression analyses.

**Results:**

Thirty-two patients (7.4%) suffered post-operative stroke. Univariate analysis identified several statistically significant risk factors in the post-operative stroke group, including pre-surgical left ventricular ejection fractions (LVEF) ≤50%, on-pump surgery, post-operative atrial fibrillation (AF), and hypotension. Multivariable analysis identified 4 independent risk factors for recurrent stroke: unstable angina (odds ratio (OR) = 2.95, 95% CI: 1.05-8.28), LVEF ≤50% (OR = 2.77, 95% CI: 1.23-6.27), AF (OR = 4.69, 95% CI: 1.89-11.63), and hypotension (OR = 2.55, 95% CI: 1.07-6.04).

**Conclusion:**

Unstable angina, LVEF ≤50%, post-operative AF, and post-operative hypotension are independent risk factors of recurrent stroke in CABG patients with a previous history of stroke.

## Introduction

Coronary artery bypass grafting (CABG) is an effective surgical therapy for coronary artery disease (CHD). However, the occurrence of stroke after CABG remains one of the most detrimental complications [1]. Due to improved surgical advances coupled with an increasing patient age, more patients with ≥1 co-morbidities are electing to undergo CABG. Included in this list of co-morbidities are an increasing number of patients with a history of stroke. Furthermore, atherosclerosis is a systemic disorder occurring throughout the vascular tree. Therefore, patients undergoing CABG are likely to show the concomitant occurrence of severe atherosclerosis in the carotid or cranial arteries, which often complicates the surgery.

The reported incidence of stroke after CABG ranges from 1 to 5% [2]. The occurrence of stroke after CABG accounts for higher hospital mortality, longer in-hospital stays, and increased costs for hospital and rehabilitative support [1]. Bucerius and colleagues have reported that patients experiencing post-operative stroke after cardiac surgery had a six-fold higher thirty-day mortality in comparison to patients without stroke [3]. The incidence of post-operative stroke in patients with a history of stroke was significantly higher than in patients with no previous history of stroke [4]. A history of stroke suggests cerebrovascular or carotid defects. In the general CABG population, the prevalence of cerebrovascular or carotid stenosis is relatively high. The reported prevalence of moderate carotid stenosis (>50% stenosis) in CABG patients is 22% [5], which results in a 3.8% stroke rate [6]. The prevalence of severe carotid stenosis (> 80% stenosis) is 8.5% [5], which results in a 14% stroke rate [7]. Therefore, cerebrovascular or carotid stenosis is considered to account for 30% of strokes associated with CABG [8]. Post-operative stroke associated with cerebrovascular disease can be divided into two principal aetiological mechanisms: hypoperfusion and thrombo-embolism. Hypoperfusion strokes arise from haemodynamic compromise distal to the carotid/cranial artery stenosis and have been associated with the patients' capacity for cerebral autoregulation [9]. Thrombo-embolic strokes are most frequently caused by thrombus formation at the site of the ulcerated atherosclerotic plaque on the carotid/cranial arteries, although the aortic arch can also be a site of thrombus formation [10].

Multiple published studies have focused on post-operative stroke, but studies on post-operative stroke in patients with a previous history of stroke have not been reported. In this study, data of 430 patients with a history of stroke that underwent CABG at our hospital were retrospectively collected and analyzed to establish the independent risk factors for this patient population.

## Materials and methods

### Patient characteristics

Four hundred and thirty-two patients with a history of stroke were selected from 6782 patients that underwent CABG at our hospital between January 2007 and July 2010. Inclusion criteria included: patients with a history of stroke that underwent either on-pump or off-pump CABG. Exclusion criteria included: patients that underwent CABG but had no previous history of stroke, patients that underwent CABG accompanied by another cardiac surgery; or patients with the history of hemorrhagic stroke prior to surgery.

### Methods

A standardized anesthetic protocol was employed using intravenous high-dose fentanyl, propofol and muscle relaxant (pipecuronium), combined with inhalation sevoflurane anesthesia during the operation. Moderate systemic hypothermia and intermittent antegrade cold blood cardioplegia were adopted for some patients (8 out of 430; 1.9%). Roller pump and membrane oxygenation was utilized in extracorporeal circulation. Acid-base balance was adjusted according to the α homeostasis principle. The database variables in our study comprised general information, including history of illness, cigarette and alcohol use, admitting diagnosis, laboratory examination, echocardiogram analysis, neuroimaging test results and surgery information (the type of surgery, number of bypass, post-operative hypotension, atrial fibrillation (AF), and stroke). All patients were in observation until their discharge. The carotid ultrasound data was limited, and was not included in our study.

### Definitions

Stroke was defined as a focal neurologic deficit and supported by a combination of residual deficits on physical examination, radiologic abnormality on computed tomography (CT) scan or magnetic resonance imaging (MRI) and documentation in the patient's medical records. The diagnosis of post-operative stroke was initially made by the surgical team and confirmed by the neurologist, based on the clinical findings and the brain imaging pictures. All imaging studies were read by staff radiologists. Early stroke was defined as stroke occurring during surgery or within 24 hours after surgery, while late stroke was defined as stroke occurring from 24 hours after surgery until day 10 post-surgery. Post-operative hypotension was defined as systolic pressure below 90 mmHg for at least 30 min, which occurred during the time period from 24 hours until 10 days after the operation.

### Statistical analysis

Statistical analyses were performed with the Statistical Package for the Social Sciences (SPSS) 11.5 software. Data were presented as mean values ± standard deviation. Univariate analysis was conducted using the chi-square test for categorical data and the t-test for measurement data. Two-tailed p-values < 0.05 were considered statistically significant. Multivariate analysis was conducted using stepwise logistic regression and considered all predictors that were significantly associated.

## Results

A total of 430 patients were included in the study. Post-operative stroke occurred in 32 patients (7.4%). Patients were divided into two groups according to the occurrence of post-operative stroke. Univariate analysis showed no significant difference in the following variables: age, sex, body mass index, history of hypertension, diabetes, lipid abnormality, atrial fibrillation, myocardial infarction, unstable angina, percutaneous transcoronary angioplasty, peripheral vascular disease, chronic obstructive pulmonary diseases, or cigarette and alcohol use. The post-operative stroke group showed a higher prevalence of EF≤50% than the control group (p = 0.006; Table 1).

**Table 1:** Pre-operative data in 430 CABG patients (n = 398 control patients and n = 32 post-operative stroke patients).

	Control Patients	Post-operative Stroke Patients	*p*
	(n = 398)	(n = 32)	
Age(years)	64.0 ± 8.6	64.7 ± 7.9	0.665
Male gender	304(76.4)	23(71.9)	0.565
Hypertension	289(72.6)	26(81.2)	0.288
Diabetes	145(36.4)	14(43.8)	0.409
Dyslipidemia	180(45.2)	13(40.6)	0.615
Pre-operative. AF	11(2.8)	1(2.9)	0.905
OMI	100(25.1)	10(31.3)	0.445
PTCA	21(5.3)	1(2.9)	0.909
PVD	9(2.3)	3(8.8)	0.073
COPD	9(2.3)	1(2.9)	0.755
Cigarette use	173(43.5)	14(43.8)	0.975
Alcohol	69(17.3)	7(20.6)	0.517
AMI	66(16.6)	4(11.8)	0.547
UAP	275(69.1)	27(84.4)	0.069
LVEF≤50%	70(17.6)	12(37.5)	0.006
BMI(kg/m^2^)	25.4 ± 2.8	24.9 ± 3.0	0.349

Data are presented as numbers (percentage). OMI: old myocardial infarction; PTCA: percutaneous transcoronary angioplasty; PVD: peripheral vascular disease; COPD: chronic obstructive pulmonary disease; AMI: acute myocardial infarction; UAP: unstable angina pectoris; LVEF: left ventricular ejection fraction; BMI: body mass index.

There were significant differences between the groups for type of surgery, post-operative hypotension, and post-operative AF (p < 0.001). No difference was found between the groups for the number of bypasses (Table 2).

**Table 2:** Post-operative data in 430 CABG patients: 398 controls vs 32 post-operative stroke

	Control Patients (n = 398)	Post-operative Stroke Patients (n = 32)	*P*
Number of bypass	3.0 ± 0.7	2.8 ± 0.8	0.242
On-pump CABG	4(1.0)	4(11.8)	<0.001
Post-operative AF	29(7.3)	11(34.4)	<0.001
Post-operative hypotentsion	53(13.3)	12(37.5)	<0.001

Data are presented as number (percentage)

Post-operative stroke occurred in four patients in the first 24 hours after operation, in 26 patients from 24 hours until 7 days after the operation, and in two patients after the seventh day. Only one out of 28 patients (3.6%) who underwent off-pump CABG experienced an early stroke event. Three out of four patients (75%) who underwent on-pump CABG experienced an early stroke event. These differences were significantly different (p < 0.05) Multivariate analysis identified four risk factors for post-operative stroke, including: unstable angina pectoris (UAP), LVEF≤50%, post-operative AF, and hypotension (Table 3). Post-operative AF occurred in 11 patients with post-operative stroke (34.3%), with stroke most often occurring within three days after the surgery. Seven of these AFs occurred before post-operative stroke occurred. Twelve patients experienced hypotension after surgery (37.5%), with seven of the hypotensive incidences occurring before stroke. For off-pump patients, there were only two risk factors identified by multivariate analysis: post-operative AF and hypotension.

**Table 3:** Multivariate regression analysis of post-operative stroke in patients with a history of stroke who underwent CABG

	Regression coefficient	SEM	*Wald*	*P*	*OR*	95%*CI*
UAP	1.083	0.526	4.237	0.040	2.952	1.053-8.277
LVEF≤50%	1.019	0.417	5.990	0.014	2.772	1.225-6.270
Post-operative AF	1.545	0.463	11.126	0.001	4.689	1.891-11.626
Post-operative hypotension	0.934	0.441	4.482	0.034	2.545	1.072-6.042

SEM: standard error of arithmetic mean; OR: odd ratios CI: confidence interval

## Discussion

In China, more elderly patients are undergoing CABG surgery to treat CHD. These patients often present with complicated co-morbidity profiles. Patients with a history of stroke undergoing CABG are high risk group for post-operative stroke [1]. Recently, to reduce the post-operative complications induced by extracorporeal circulation, off-pump CABG has been extensively adopted. In our study, most of the surgeries were off-pump CABG; only 8 of 430 were on-pump CABG (1.9%).

Post-operative stroke occurred in 32 patients in our study (7.4%). The events can be divided into early stroke and late stroke, according to the time that stroke occurred after surgery. In our study, four patients suffered early stroke, and three of them underwent on-pump CABG. Peel and colleagues found that on-pump CABG showed a higher incidence of early stroke, and off-pump CABG showed a higher incidence of late stroke, which is similar with the results of our study [11].

The risk factors of post-operative stroke can be categorized into pre-operative, intra-operative, and post-operative factors. Pre-operative factors include advanced age, atherosclerosis in ascending aorta, unstable angina, hypertension and history of stroke; Intra-operative factors include the endurance of extracorporeal circulation and aorta clamping, or operation type. Post-operative factors include AF, micro-embolism detachment, and hypotension [12,13]. Our study selected patients with a history of stroke who were the high risk group for post-operative stroke. We identified risk factors for recurrent stroke, including unstable angina, LVEF≤50%, post-operative AF, and hypotension. Extracorporeal circulation was a statistically significant variable for post-operative stroke in univariate analysis, but was excluded in the multivariate analysis. The limited number of on-pump CABG may account for the lack of significance seen in the on-pump CABG group.

The incidence of post-operative AF is 5%-40%, and in off-pump CABG it has been shown to occur at incidences ranging from 0 to 26% [14,15]. While AF most often occurs within 2-3 days after surgery, most of these occurrences are self-limited [16,17]. There are many determinants for post-operative AF, including increasing age, male gender, the incidence of right coronary artery stenosis, excessive manipulation of the heart during surgery and electrolyte imbalance. The correlation between cerebral embolism and AF has been well established. Many studies have proved that post-operative AF is the risk factor of post-operative stroke. Lahtinen and colleagues reported that the average interval from first AF to post-operative stroke was 21.3 hours, and post-operative stroke patients suffered average 2.5 postoperative AF occurrences before stroke [18]. In our study, eleven of 32 patients with post-operative stroke (35.3%) suffered postoperative AF, with most of the AF incidences occurring within three days after stroke, and seven of the incidences occurring before post-operative stroke.

Currently, the interaction between embolism and hypoperfusion is generally considered to be a major cause of post-operative stroke. Hypoperfusion may contribute to embolus retention. Several studies have found multiple emboli in the cortical watershed of patients who died after cardiac surgery, which confirms the synergistic effect of hypoperfusion and embolism on postoperative stroke. Our study suggests that unstable angina, LVEF≤50%, and hypotension are risk factors of post-operative recurrent stroke. All of these factors decrease brain perfusion, leading to stroke. Because the cerebral arteriosclerosis of patients with a history of stroke are more severe than patients without stroke, most of these patients progress to artery stenosis, where a relatively higher blood pressure is needed to maintain cerebral perfusion. When mean arterial pressure decreases 10 mmHg, the incidence of cortical watershed infarction increases by four-fold [19].

In pre-infarction period that occurs before stroke, cerebral circulation reserve cannot compensate for the hypoperfusion status, and this decompensation stage may last for several years. This stage often manifests with headache and difficulty in speaking. [20]. Patients with post-operative stroke may have been in the pre-infarction decompensated stage before surgery. Because of this potential complication, a pre-operative cerebral haemodynamic evaluation, including transcranial doppler sonography, vascular ultrasound, and CT perfusion imaging for high risk patients is important for the prevention of postoperative stroke. [21,22].

After excluding on-pump patients, multivariate analysis eliminated both UAP and LVEF≤50% from the list of risk factors for post-operative stroke. This suggests that myocardial ischemia and cardiac function may play an important role in post-operative recurrent stroke for on-pump patients, while it influences less those patients that are off-pump. However, due to the limited number of on-pump patients, this concept should be studied in a larger clinical trial.

In conclusion, patients with a history of stroke who undergo CABG are often relatively older and have more complicated co-morbidities. Since our study is a retrospective study, information such as the extent of intracranial and extracranial arteriostenosis is lacking. Future studies, especially prospective studies, are needed to determine the optimum therapeutic strategy for preventing the occurrence of post-operative stroke in patients with a history of stroke. Based on our results, strategies that focus on patients with unstable angina, LVEF≤50% and hypotension may provide improved outcomes.

## Competing interests

The authors declare that they have no competing interests.

## Authors' contributions

LC was responsible for the study design, data collection, statistical analysis, data interpretation, and drafting the manuscript. QL was responsible for data collection. QB was responsible for drafting the manuscript and revising it critically for important content. QJY was responsible for the study design and the final approval of article. All authors read and approved the final manuscript.
